# Aminodi(hetero)arylamines in the Thieno[3,2-*b*]pyridine Series: Synthesis, Effects in Human Tumor Cells Growth, Cell Cycle Analysis, Apoptosis and Evaluation of Toxicity Using Non-Tumor Cells

**DOI:** 10.3390/molecules17043834

**Published:** 2012-03-28

**Authors:** Ricardo C. Calhelha, Isabel C. F. R. Ferreira, Daniela Peixoto, Rui M. V. Abreu, Luís A. Vale-Silva, Eugénia Pinto, Raquel T. Lima, M. Inês Alvelos, M. Helena Vasconcelos, Maria-João R. P. Queiroz

**Affiliations:** 1 Center of Chemistry, University of Minho, Campus de Gualtar 4710-057 Braga, Portugal; 2 CIMO-ESA, Instituto Politécnico de Bragança, Campus de Sta Apolónia, Apartado 1172, 5301-855 Bragança, Portugal; 3 Department of Biological Sciences, Faculty of Pharmacy of the University of Porto, Rua de Jorge Viterbo Ferreira n.° 228, 4050-313 Porto, Portugal; 4 CEQUIMED-UP, Research Center of Medicinal Chemistry, University of Porto, Rua de Jorge Viterbo Ferreira n.° 228, 4050-313 Porto, Portugal; 5 Cancer Drug Resistance Group, IPATIMUP-Institute of Molecular Pathology and Immunology of the University of Porto, Rua Dr. Roberto Frias s/n, 4200-465 Porto, Portugal

**Keywords:** thieno[3,2-*b*]pyridines, aminodi(hetero)arylamines, Buchwald-Hartwig C-N coupling, antitumoral activity, toxicity, cell cycle, apoptosis

## Abstract

Three aminodi(hetero)arylamines were prepared *via* a palladium-catalyzed C-N Buchwald-Hartwig coupling of methyl 3-aminothieno[3,2-*b*]pyridine-2-carboxylate with different bromonitrobenzenes, followed by reduction of the nitro groups of the coupling products to the corresponding amino compounds. The aminodi(hetero)arylamines thus obtained were evaluated for their growth inhibitory effect on four human tumor cell lines MCF-7 (breast adenocarcinoma), A375-C5 (melanoma), NCI-H460 (non-small cell lung cancer) and HepG_2_ (hepatocellular carcinoma). The toxicity to non-tumor cells was also evaluated using a porcine liver primary cell culture (PLP1), established by us. The aminodi(hetero)arylamine with the NH_2_ group in the ortho position and an OMe group in the *para* position to the NH of the di(hetero)arylamine, is the most promising compound giving the lowest GI_50_ values (1.30–1.63 µM) in all the tested human tumor cell lines, presenting no toxicity to PLP1 at those concentrations. The effect of this compound on the cell cycle and induction of apoptosis was analyzed in the NCI-H460 cell line. It was observed that it altered the cell cycle profile causing a decrease in the percentage of cells in the G0/G1 phase and an increase of the apoptosis levels.

## 1. Introduction

Despite the great amount of research and rapid development of new therapies seen during the past decade, cancer continues to be a leading cause of death worldwide. It is estimated that by 2020 approximately 15 million new cancer cases will be diagnosed and 12 million patients will die every year [1]. Although conventional anti-cancer therapies in current clinical use (such as anti-hormonal therapy, radiotherapy and chemotherapy) improve patient survival, the resulting drug toxicity and severe side effects are still causing high failure rates of cure. In addition, when organ-confined tumors advance to locally invasive or metastatic stages, this usually associates to resistance to conventional therapies, disease relapse, and to patient death within a short period of time [2].

Several thienopyridines have already been described as inhibitors of cell proliferation in *in vitro* tumoral cell line assays [3,4], highlighting the interest of studying their anti-tumoral activity. Our research group has also synthesized various methyl 3-aminothieno[3,2-*b*]pyridine-2-carboxylate derivatives functionalized at the pyridine ring by Pd-catalyzed C-N (Buchwald-Hartwig) and C-C (Suzuki and Sonogashira) couplings and some of them presented cell growth inhibitory activity in tumor cell lines [5,6,7]. Among the 6-(hetero)arylaminothieno[3,2-*b*]pyridine derivatives obtained by C-N coupling the most promising compounds bear a benzothiazole (GI_50_ 3.5–6.4 µM) or an indole (GI_50_ 15.8–18.1 µM) moiety [5]. 

In the present work, three aminodi(hetero)arylamines in the thieno[3,2-*b*]pyridine series functionalized at the thiophene ring were synthesized from the corresponding nitro compounds. The aminodi(hetero)arylamines were evaluated for their *in vitro* growth inhibitory effect on four human tumor cell lines [MCF-7 (breast adenocarcinoma), NCI-H460 (non-small cell lung cancer), A375-C5 (melanoma) and HepG_2_ (hepatocellular carcinoma)] and also on the non-tumor primary cells PLP1 (porcine liver primary cell culture) established by us. For the most active and less toxic compound, its effects on cell cycle and induction of apoptosis were evaluated using the NCI-H460 cell line.

## 2. Results and Discussion

### 2.1. Synthesis

**Scheme 1:**
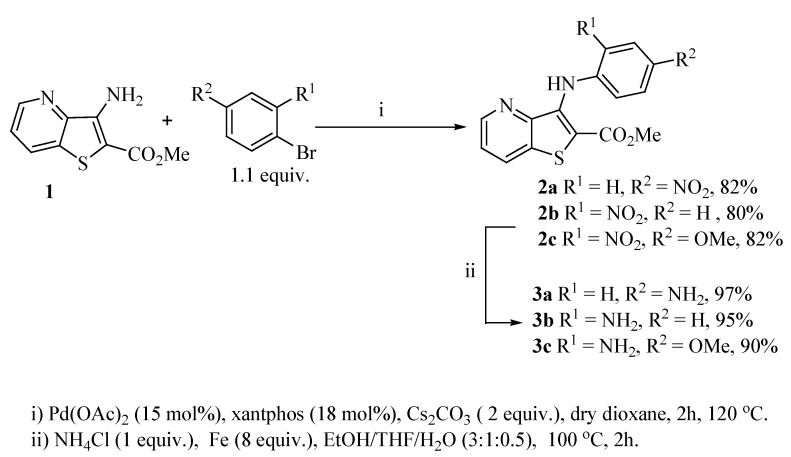
Synthesis of di(hetero)arylnitro compounds **2a**–**c** by Buchwald-Hartwig C-N coupling and their reduction to the aminodi(hetero)arylamines **3a**–**c**.

Reacting methyl 3-aminothieno[3,2-*b*]pyridine-2-carboxylate (**1**) [8] with different bromo-nitrobenzenes under Pd-catalyzed Buchwald-Hartwig C-N coupling conditions (i) using xantphos as the ligand and Cs_2_CO_3_ as the base, the di(hetero)arylnitro compounds **2a**–**c** were obtained in high yields. These conditions were already used by others [9] and by some of us [10] for the C-N coupling of deactivated amines, with good results. The nitro compounds **2a**–**c** were reduced in almost quantitative yield to the corresponding aminodi(hetero)arylamines **3a**–**c** using conditions; (ii) [11] depicted in Scheme 1. The synthesis of compounds **2a**, **2b** and **3a**, **3b** was already schematically presented by us in an earlier short communication [12], but the experimental procedure of their synthesis and their complete characterization are described here for the first time, together with compounds **2c** and **3c**.

### 2.2. Cell Growth Inhibitory Activity and Toxicity to Non-Tumor Cells

The effects of the aminodi(hetero)arylamines in the growth of four human tumour cell lines (MCF-7, A375-C5, NCI-H460 and HepG_2_) were evaluated using the SRB assay. The results, represented as the concentrations that caused 50% of cell growth inhibition (GI_50_), are summarized in Table 1. Doxorubicin and ellipticine were used as positive controls. Furthermore, to investigate the possible toxicity of the compounds, the *in vitro* cell growth inhibition assay (SRB) was also performed using porcine liver non-tumor cells (PLP1) established by us.

From the analysis of Table 1 it is possible to conclude that the compound **3c** with the amino group in the *ortho* position and a methoxy group in the *para* position presented the lowest GI_50_ values (<2 µM) in all the tested human tumour cell lines. Moreover, those values were always lower than the GI_50_ value found in the toxicity assay, when using the porcine liver non-tumor cells (PLP1). It is also observed that the presence of the amino group in the *ortho* position to the NH in compound **3b** significantly decreased the GI_50_ values when compared to the presence of the amino group in the *para* position in compound **3a**.

**Table 1:** GI_50_ values^a^ (μM) for the aminodi(hetero)arylamines **3a**–**c** and for the controls doxorubicin and ellipticine (mean ± SD; n = 3).

	MCF-7	A375-C5	NCI-H460	HepG2	PLP1
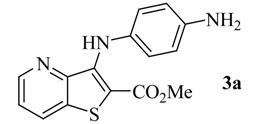	>125	111.80 ± 5.00	>125	>125	^>125^
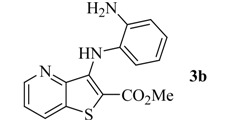	33.80 ± 1.70	26.00 ± 2.30	31.30 ± 2.90	99.26 ± 0.92	^61.27 ± 1.83 ^
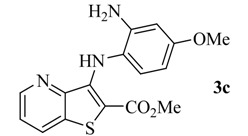	1.40 ± 0.20	1.30 ± 0.10	1.40 ± 0.40	1.63 ± 0.09	^12.49 ± 0.09^
**Doxorubicin**	0.04 ± 0.01	0.13 ± 0.01	0.09 ± 0.01	---	---
**Ellipticine**	---	---	---	0.80 ± 0.05	^4.19 ± 0.08^

^a^ Results represent the GI_50_ concentrations (concentrations that were able to cause 50% of cell growth inhibition) determined following a 48h continuous treatment.

**Figure 1:**
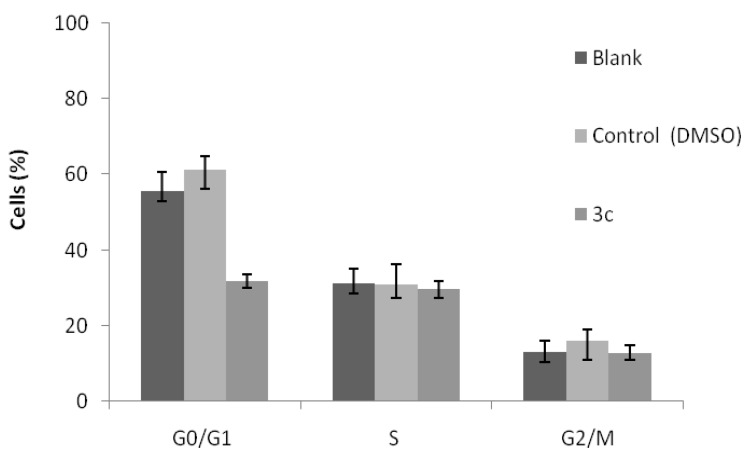
Cell cycle analysis of NCI-H460 cells treated with compound **3c** at its GI_50_ concentration (1.4 μM). Untreated cells (blank) and compound vehicle (DMSO) were used as controls. Results are the mean ± SEM of three independent experiments.

The effect of the most promising compound **3c** in the cell cycle profile was then analyzed in the NCI-H460 cells. The results, presented in Figure 1, show that this compound caused a significant decrease in the percentage of cells in the G0/G1 phase of the cell cycle. 

In addition the compound **3c **significantly increased the % of cells in the sub-G1 peak (data not shown), which is indicative of apoptosis. In order to confirm this, flow cytometry analysis was carried out following Annexin V/Propidium iodide staining. Results clearly showed that compound **3c** statistically increased the percentage of apoptotic cells, from 6.5 ± 1.1% in blank cells (or 4.2 ± 0.4% in treated cells with DMSO) to 36.6 ± 4.8% in treated cells with compound **3c**. 

## 3. Experimental

### 3.1. Synthesis

Melting points (°C) were determined in a Stuart SMP3 and are uncorrected. ^1^H- and ^13^C-NMR spectra were recorded on a Varian Unity Plus at 300 and 75.4 MHz, respectively or on a Bruker Avance III at 400 and 100.6 MHz, respectively. Two dimensional ^1^H-^13^C correlations were performed to attribute some signals. HRMS using EI was performed by the mass spectrometry service of the University of Vigo, C.A.C.T.I., Spain. The reactions were monitored by thin layer chromatography (TLC) using Macherey-Nagel pre-coated aluminium silica gel 60 sheets (0.20 mm) with UV254 indicator. Dry-flash cromatography was performed on Panreac, Silica Gel 60, 40–63 microns. Ether refers to diethylether. Xantphos refers to 8,9-dimethyl-4,5-bis(diphenylphosphane)xanthene.

#### 3.1.1. General Procedure for the Synthesis of Nitrodiarylamines **2a–c**

A dry Schlenk tube was charged under Ar with dry dioxane (3 mL), the bromonitrobenzene (1.1 equiv.), Pd(OAc)_2_ (15 mol%), Xantphos (18 mol%), Cs_2_CO_3_ (2 equiv.) and the methyl 3-aminothieno[3,2-*b*]pyridine-2-carboxylate **(1)**, and the mixture was heated under Ar at 120 °C for 2 h. After cooling water (5 mL) and ethyl acetate (5 mL) were added. The phases were separated and the aqueous phase was extracted with more ethyl acetate (2 × 5 mL). The organic phase was dried (MgSO_4_) and filtered. Removal of the solvent gave a brown solid which was submitted to dry flash using ether and a solid was obtained.

*Methyl 3-(4-nitrophenylamino)thieno[3,2-b]pyridine-2-carboxylate ***(2a)**: From thienopyridine **1** (150 mg, 0.724 mmol) and 1-bromo-4-nitrobenzene (161 mg, 0.796 mmol), compound **2a** was isolated as a brownish solid (195 mg, 82%), m.p. 203–205 °C. ^1^H-NMR (400 MHz, DMSO-*d_6_*): δ = 3.96 (s, 3H, OMe), 7.08 (d, *J* = 9.2 Hz, 2H, 2′ and 6′-H), 7.43 (dd, *J* = 8.2 and 4.2 Hz 1H, 6-H), 8.13 (d, *J* = 9.2 Hz, 2H, 3′ and 5′-H), 8.19 (dd, *J* = 8.2 and 1.6 Hz, 1H, 7-H), 8.67 (dd, *J* = 4.2 and 1.6 Hz, 1H, 5-H), 8.76 (br s, 1H, NH) ppm. ^13^C-NMR (100.6 MHz, DMSO-*d_6_*): δ = 52.5 (OMe), 113.8 (C-2), 118.2 (2′ and 6′-CH), 122.1 (6-CH), 124.9 (3′ and 5′-CH), 131.5 (7-CH), 134.2 (C), 141.7 (C), 141.8 (C), 147.2 (5-CH), 147.6 (C-3), 148.0 (C-1’), 164.5 (C=O) ppm. HRMS (EI) Calcd. for C_15_H_11_N_3_O_4_S [M^+^] 329.0470; found 329.0479. 

*Methyl 3-(2-nitrophenylamino)thieno[3,2-b]pyridine-2-carboxylate ***(2b)**: From thienopyridine **1** (150 mg, 0.724 mmol) and 1-bromo-2-nitrobenzene (161 mg, 0.796 mmol), compound **2b** was isolated as a reddish solid (190 mg, 80%), m.p. 202–204 °C. ^1^H-NMR (400 MHz, DMSO-*d_6_*): δ = 3.97 (s, 3H, OMe), 6.93–7.00 (m, 2H, 6′ and 5′-H), 7.32–7.37 (m, 1H, 4′-H), 7.42 (dd, *J* = 8.2 and 4.4 Hz, 1H, 6-H), 8.19-8.22 (m, 2H, 3′ and 7-H), 8.67 (dd, 1H, *J* = 4.4 and 1.6 Hz, 5-H), 10.59 (br s, 1H, NH) ppm. ^13^C-NMR (100.6 MHz, DMSO-*d_6_*): δ = 52.7 (OMe), 118.6 (C-2), 119.9 (5′-CH), 120.1 (6′-CH), 121.8 (6-CH), 125.9 (3′-CH), 131.4 (7-CH), 133.9 (4′-CH), 134.00 (C-1′), 135.9 (C), 139.2 (C), 139.3 (C), 147.4 (5-CH), 148.3 (C), 163.3 (C=O) ppm. HRMS (EI) Calcd. for C_15_H_11_N_3_O_4_S [M^+^] 329.0470; found 329.0478. 

*Methyl 3-(4-methoxy-2-nitrophenylamino)thieno[3,2-b]pyridine-2-carboxylate ***(2c)**: From thieno-pyridine **1** (150 mg, 0.724 mmol) and 4-bromo-3-nitroanisole (185 mg, 0.796 mmol), compound **2c** was isolated as a reddish solid (220 mg, 82%), m.p. 213–214 °C. ^1^H-NMR (400 MHz, DMSO-*d_6_*): δ = 3.82 (s, 3H, OMe), 3.87 (s, 3H, OMe), 7.02 (d, *J* = 9.2 Hz, 1H, 6′-H), 7.14 (dd, *J* = 9.2 and 2.8 Hz, 1H, 5′-H), 7.53–7.58 (m, 2H, 3′ and 6-H), 8.55 (dd, *J* = 8.4 and 1.6 Hz, 1H, 7-H), 8.60 (dd, *J* = 4.4 and 1.6 Hz, 1H, 5-H), 9.72 (br s, 1H, NH) ppm. ^13^C-NMR (100.6 MHz, DMSO-*d_6_*): δ = 52.5 (OMe), 55.8 (OMe), 107.2 (3′-CH), 114.3 (C-2′), 122.5 (6-CH), 122.9 (5′-CH), 123.5 (6′-CH), 132.1 (C), 132.4 (7-CH), 133.5 (C), 136.8 (C-1′), 139.9 (C), 147.1 (C), 147.4 (5-CH), 153.2 (C-2), 163.3 (C=O) ppm. HRMS (EI) Calcd. for C_16_H_13_N_3_O_5_S [M^+^] 359.0576; found 359.0559. 

#### 3.1.2. General Procedure for the Synthesis of Aminodiarylamines **3a–c**

In a round bottom flask with 4.5 mL EtOH/THF/H_2_O (3:1:0.5), compounds **2a–c**, NH_4_Cl (1 equiv.), Fe (8 equiv.) were heated at 100 °C for 2 h. After cooling the mixture was evaporated to give a dark solid. This was submitted to dry-flash using ether and the product was obtained as a yellow or greenish solid.

*Methyl 3-(4-aminophenylamino)thieno[3,2-b]pyridine-2-carboxylate ***(3a)**: From nitrodiarylamine **2a** (100 mg, 0.304 mmol) compound **3a** was isolated as a greenish solid (88.0 mg, 97%), m.p. 200–202 °C. ^1^H-NMR (300 MHz, DMSO-*d_6_*): δ = 3.77 (s, 3H, OMe), 4.84 (br s, 2H, NH_2_), 6.45 (d, *J* = 8.4 Hz, 2H, 3′ and 5′-H), 6.77 (d, *J* = 8.4 Hz, 2H, 2′ and 6′-H), 7.46 (dd *J* = 8.2 and 4.2 Hz, 1H, 6-H), 8.39 (dd, *J* = 8.2 and 1.6 Hz, 1H, 7-H), 8.51 (dd, *J* = 4.2 and 1.6 Hz, 1H, 5-H), 8.60 (br s, 1H, NH) ppm. ^13^C-NMR (75.4 MHz, DMSO-*d_6_*,): δ = 51.9 (OMe), 103.6 (C), 113.7 (3′ and 5′-CH), 122.3 (6-CH), 123.0 (2′ and 6′-CH), 130.9 (C), 131.8 (7-CH), 133.7 (C), 144.9 (C-4′), 145.3 (C), 146.4 (5-CH), 147.2 (C), 164.4 (C=O) ppm. HRMS (EI) Calcd. for C_15_H_13_N_3_O_2_S [M^+^] 299.0728; found 299.0732.

*Methyl 3-(2-aminophenylamino)thieno[3,2-b]pyridine-2-carboxylate ***(3b)**: From nitrodiarylamine **2b** (100 mg, 0.304 mmol) compound **3b** was isolated as a greenish solid (86.0 mg, 95%), m.p. 204–206 °C. ^1^H-NMR (400 MHz, DMSO-*d_6_*): δ = 3.79 (s, 3H, OMe), 4.97 (br s, 2H, NH_2_), 6.39 (m, 1H, ArH), 6.63–6.83 (m, 3H, ArH), 7.48 (m, 1H, 6-H), 8.12 (br s, 1H, NH), 8.42–8.52 (m, 2H, 7 and 5-H) ppm. ^13^C-NMR (100.6 MHz, DMSO-*d_6_*): δ = 52.0 (OMe), 105.9 (C-2), 114.9 (CH), 115.9 (CH), 122.2 (CH), 122.3 (6-CH), 124.1 (CH), 127.9 (C), 131.8 (7-CH), 133.6 (C), 141.2 (C), 145.2 (C), 146.7 (5-CH), 147.2 (C), 164.2 (C=O) ppm. HRMS (EI) Calcd. for C_15_H_13_N_3_O_2_S [M^+^] 299.0728; found 299.0726.

*Methyl 3-(2-amino-4-methoxhyphenylamino)thieno[3,2-b]pyridine-2-carboxylate ***(3c)**: From nitro-diarylamine **2c** (100 mg, 0.278 mmol) compound **3c** was isolated as a greenish solid (83.0 mg, 90%), m.p. 172–174 °C. ^1^H-NMR (400 MHz, DMSO-*d_6_*): δ = 3.65 (s, 3H, OMe), 3.79 (s, 3H, OMe), 5.00 (br s, 2H, NH_2_), 5.99 (dd, *J* = 8.8 and 2.4 Hz, 1H, 5′-H), 6.31 (d, *J* = 2.4 Hz, 1H, 3′-H), 6.61 (d, *J* = 8.8 Hz, 1H, 6′-H), 7.43 (dd, *J *= 8.2 and 4.4 Hz, 1H, 6-H), 8.09 (br s, 1H, NH), 8.38 (dd, *J* = 8.2 and 1.6 Hz, 1H, 7-H), 8.47 (dd, *J* = 4.4 and 1.6 Hz, 1H, 5-H) ppm. ^13^C-NMR (100.6 MHz, DMSO-*d_6_*): δ = 51.9 (OMe), 54.8 (OMe), 100.0 (3′-CH), 101.2 (5′-CH), 103.4 (C), 120.9 (C), 122.2 (6-CH), 124.9 (6′-CH), 131.7 (7-CH), 133.68 (C), 143.7 (C), 146.5 (C), 146.5 (5-CH), 147.0 (C), 157.2 (C-4’), 164.6 (C=O) ppm. HRMS (EI) Calcd. for C_16_H_15_N_3_O_3_S [M^+^] 329.0834; found 329.0827.

### 3.2. Cell Growth Inhibitory Activity and Toxicity to Non-Tumor Cells

#### 3.2.1. Standards and Reagents

DMSO (dimethyl sulfoxide) was analytical grade from Fisher Scientific (Paris, France). Fetal bovine serum (FBS), L-glutamine, hank’s balanced salt solution (HBSS), trypsin-EDTA (ethylenediaminetetraacetic acid), penincillin/streptomycin solution (100 U/mL and 100 mg/mL, respectively) and RNAse A were from Gibco Invitrogen Co. (Paisley, UK). RPMI-1640 medium was from Cambrex (East Rutherford, NJ, USA) and DMEM medium was from Hyclone (Logan, UT, USA). Acetic acid, ellipticine, doxorubicin, sulforhodamine B (SRB), trypan blue, trichloroacetic acid (TCA), Tris and propidium iodide were from Sigma Chemical Co. (St. Louis, MO, USA). Water was treated in a Mili-Q water purification system (TGI Pure Water Systems, Greenville, SC, USA).

#### 3.2.2. Solutions of the Compounds

Stock solutions of the tested aminodiarylamines were prepared in DMSO and kept at −20 °C. Appropriate dilutions were prepared in the corresponding solvent prior to the assays. The effect of DMSO on the growth of the cell lines was evaluated by treating cells with the maximum concentration of DMSO used in the assays (0.25%). No influence was found (data not shown).

#### 3.2.3. Cell Culture of Cell Lines and of Porcine liver Primary Cells

Four human tumor cell lines, MCF-7 (breast adenocarcinoma), A375-C5 (melanoma), NCI-H460 (non-small cell lung cancer) and HepG2 (hepatocellular carcinoma) were used. MCF-7, A375-C5 and HepG2 were obtained from the European Collection of Cell Cultures (ECACC, Salisbury, UK) and NCI-H460 was kindly provided by the National Cancer Institute (NCI, Bethesda, MD, USA). Cells were routinely maintained as adherent cell cultures in RPMI-1640 medium supplemented with 5% FBS (MCF-7, NCI-H460 and A375-C5 cells) or in DMEM supplemented with 10% FBS, 2 mM glutamine, 100 U/mL penicillin and 100 mg/mL streptomycin (HepG_2_ cells), at 37 °C in a humidified atmosphere containing 5% CO_2_.

Porcine liver primary cell culture (PLP1) was prepared from freshly harvested porcine liver, obtained from a local slaughter house. Briefly, the liver tissue was rinsed in HBSS solution containing 100 U/mL penicillin, 100 µg/mL streptomycin and divided into 1 × 1 mm^3^ explants. Some of these explants were placed in 25 cm^2^ tissue flasks in DMEM medium supplemented with 10% FBS, 2 mM nonessential amino acids, 100 U/mL penicillin and 100 mg/mL streptomycin. This cell culture was maintained according to the procedure described previously by us [13].

#### 3.2.4. Cell Growth Inhibition Assay in Tumour Cell Lines and Primary Porcine Liver Cells

The effects of the compounds on the *in vitro* growth of human tumor cell lines and non-tumor porcine liver primary cells were evaluated according to the procedure adopted by the NCI (USA) in their “*In vitro* Anticancer Drug Discovery Screen”, using the SRB assay to assess cell growth [5,6,7].

For the cell lines, exponentially growing cells were obtained by plating 1.5 × 10^5^ cells/mL for MCF-7, 0.75 × 10^5^ cells/mL for A375-C5 and NCI-H460, and 5.0 × 10^5^ cells/mL for HepG2, followed by 24 h of incubation. For the primary porcine liver cells, cells were incubated in 96-well plates at a density of 5.0 × 10^5^ cells/mL, and cultivated in DMEM medium with 10% FBS, 100 U/mL penicillin and 100 mg/mL streptomycin. 

Following a 24 h period to allow cells to adhere, cells were treated for 48 h with six serial dilutions of each test compound, starting from a maximum concentration of 125 µM. Following the 48 h treatment period, adherent cells were fixed *in situ*, washed and stained with SRB. The bound stain was solubilized and the absorbance was measured at 492 nm in the case of MCF-7, NCI-H460 and A375-C5 cells and at 515 nm in the case od HepG2 and PLP1 primary cells, in a plate reader (Biotek Instruments Inc., Powerwave XS, Bedfordshire, UK). Dose response curves were obtained and the concentration of the diarylamine that inhibited 50% of the net cell growth (GI_50_) was calculated as described elsewhere [6,7,8]. Doxorubicin and ellipticine were used as positive controls of cell growth inhibition.

#### 3.2.5. Analysis of Cell Cycle Distribution Profile

NCI-H460 cells were plated in 6-well plates at a final density of 1.5 × 10^5^ cells/well and incubated at 37 °C for 24 h. Cells were then treated with compound **3c** at its GI_50_ concentration (1.4 µM). As controls, cells were also incubated with medium only (blank) and with the compound’s vehicle (DMSO). Following 48 h of treatment, cells were fixed overnight in ice-cold 70% ethanol and re-suspended in PBS containing 0.1 mg/mL RNase A and 5 µg/mL propidium iodide. Cellular DNA content was analysed by flow cytometry using an Epics XL-MCL Coulter Flow cytometer (Brea, CA, USA) plotting at least 20,000 events *per* sample, as previously described [6,7]. The analysis of cell cycle distribution was subsequently performed using the FlowJo 7.2 software (Tree Star, Inc., Ashland, USA) after cell debris and aggregates exclusion.

#### 3.2.6. Analysis of Apoptosis

Following 48 h treatment of NCI-H460 cells with compound **3c**, as described above, the levels of apoptosis were analysed by flow cytometry using the Human Annexin V-FITC/PI apoptosis kit (Bender MedSystems, Vienna, Austria), according to the manufacturer’s instructions. Flow cytometry was carried out using an Epics XL-MCL Coulter flow cytometer plotting at least 20,000 events per sample, as previously described [6,7]. The data was analysed using the FlowJo 7.2 software (Tree Star, Inc.).

## 4. Conclusions

From the three aminodi(hetero)arylamines synthesized, compound **3c**, with the NH_2_ group in *ortho* and an OMe group in *para* to the NH of the di(heteroarylamine, is the most promising antitumoral compound presenting the lowest GI_50_ values in all the human tumor cell lines studied. This compound did not show toxicity towards porcine liver non-tumor cells at those concentrations. In addition, all the aminodi(hetero)arylamines **3a–c** presented lower toxicity to non-tumor cells than the positive control, ellipticine (GI_50_ = 4 µM). Moreover, compound **3c** caused a change in the cell cycle profile of the NCI-H460 cells, leading to a decrease in the percentage of cells in the G0/G1 phase of the cell cycle and caused an increase in the percentage of NCI-H460 apoptotic cells.
